# Comparison and Analysis of Antibiotic Consumption in Two Italian Hospital Settings in Relation to the Fight of Antimicrobial Resistance

**DOI:** 10.3390/ph17020183

**Published:** 2024-01-30

**Authors:** Francesco Ferrara, Roberta Pasquinucci, Maurizio Capuozzo, Giacomo Polito, Gabriele Bagaglini, Marcello Vaccaro, Adriana Coluccia, Roberto Langella, Ugo Trama, Eduardo Nava, Andrea Zovi

**Affiliations:** 1 Pharmacy Unit, ASL Napoli 3 Sud, 80035 Naples, Italy; r.pasquinucci@aslnapoli3sud.it (R.P.); m.capuozzo@aslnapoli3sud.it (M.C.); e.nava@aslnapoli3sud.it (E.N.); 2 Pharmacy Unit, Pharmacy Department, Policlinico Umberto I, 00161 Rome, Italy; g.polito@policlinicoumberto1.it (G.P.); marcello.vac@gmail.com (M.V.); coluccia.ad@gmail.com (A.C.); 3 Pharmacy Unit, ASL Latina, 04100 Latina, Italy; dott.gabrielebagaglini@hotmail.it; 4 Italian Society of Hospital Pharmacy (SIFO), SIFO Secretariat of the Lombardy Region, 20159 Milan, Italy; roberto.langella87@gmail.com; 5 Health Protection and Coordination of the Campania Regional Health System, 80143 Naples, Italy; ugo.trama@regione.campania.it; 6 Ministry of Health, 00144 Rome, Italy; zovi.andrea@gmail.com

**Keywords:** antimicrobial resistance, antibiotics, appropriateness, consumption, assessment, defined daily dose

## Abstract

**Introduction**: The emergence and spread of drug-resistant pathogens due to the improper use of antibiotics have become increasingly apparent in recent years. **Objective**: This retrospective comparative analysis aimed to assess and compare antibiotic prescription trends in Italy across two different regions based on geographic area and healthcare structure. One region represents a large hospital institution, while the other represents a populous local Italian health agency. The study also examined the impact of documented antibiotic stewardship programs and efforts to promote responsible antibiotic use at all levels, in alignment with international goals. Antibiotic consumption data were collected from the Umberto I Polyclinic Hospital and the ASL Napoli 3 South Local Health Agency. **Methods**: To compare consumption between regions, a standardized comparison using the Defined Daily Dose (DDD) was employed. The internal management system of each healthcare facility records all prescriptions and drug dispensations, and these data were extrapolated for this retrospective study. **Results**: A comparative assessment between the first half of 2022 and 2023 (January–June) highlighted a significant increase in beta-lactam antibiotic consumption, showing a twofold rise compared to the previous year’s term. Regarding prescription averages, there was a noticeable increase of +29.00% in hospitalizations and +28.00% in hospital discharges within the ASL Napoli 3 South. Conversely, at Policlinico Umberto I, there was a marginal increase of +1.60% in hospitalizations and a decrease of −7.40% in hospital discharges. **Conclusions**: The study offers valuable insights into expenditure patterns and antibiotic consumption, underscoring the need for enhanced prescribing practices and awareness campaigns to address the issue of antibiotic resistance. The findings stress the importance of implementing international guidelines to combat the growing threat of antibiotic resistance and ensure the effective management of infectious diseases.

## 1. Introduction

Resistant to antimicrobials (AMR), counted among the top three hazards to human well-being, is acknowledged as a global apprehension with substantial epidemiological and economic repercussions, notwithstanding the unrelenting surveillance exertions worldwide, in the European Union (EU), and the European Economic Area (EEA) [1]. Every year, approximately 35,000 fatalities transpire in the EU/EEA, where one-third of these are ascribed to infections triggered by bacteria that resist antibiotics, and 70% are associated with infections acquired in healthcare settings [2]. Recognizing the interdependency of human health, animal health, and ecosystem health, a worldwide “One Health” tactic deals with AMR, promoting collaboration between nations and sectors to advocate for the circumspect application of antibiotics in human, veterinary, and environmental domains [3]. Recognizing antibiotic resistance as a healthcare priority, member states of the EU were enjoined in the Council of the European Union on June 17, 2016, to devise a national strategy against antimicrobial resistance by the middle of 2017. This strategy is grounded in the “One Health” approach and aligns with the goals of the global action plan of the World Health Organization (WHO) [4]. At the national echelon, the National Strategy against Antimicrobial Resistance (PNCAR) 2017–2020, extended through 2021, and subsequently the National Plan to Counteract Antibiotic Resistance (PNCAR) 2022–2025 were sanctioned, rooted in inclusive governance. The strategy is organized around four horizontal spheres endorsing pivotal themes (Training, Information—Communication and Transparency, Research—Innovation and Bioethics, National and International Cooperation) and three vertical columns concentrating on interventions for the prevention and control of antibiotic resistance in human, animal, and environmental fields [5]. Pivotal alterations in the modern national strategy encompass a more comprehensive application of the One Health approach; heightened stress on communication, transparency, and bioethical facets; augmented integration between human, veterinary, and environmental sectors; and fortified surveillance of antimicrobials and activities for preventing and controlling infections. In spite of a dwindling pattern, the utilization of antibiotics in Italy remains beyond the European average in both the human and veterinary fields, with marked heterogeneity among regions in Italy [6]. The aim of this study is to evaluate the trend of antibiotic prescriptions in two Italian countries that differ in geographical area to compare the data collected and discuss the impact of documented antibiotic stewardship programs and efforts to promote a conscious and rational use of antibiotics in the field at all levels, in line with the objectives set by the international community. An all-Italian direct comparison between two different regions and between a hospital and a local health authority can provide new and important evidence, which can be useful to enrich the existing literature, on the national situation of an industrialized European country with one of the highest rates of bacterial resistance. This document will serve to make all healthcare personnel aware of the measures taken so far and those still needed to improve antibiotic consumption and prescription. Pivotal factors jeopardizing antibiotic resistance, leading to escalated management expenses in hospitals, include frequent and often improper application of broad-spectrum antibiotics, at times administered at less-than-optimal concentrations [7,8,9]. Consequently, vigilance over the usage of antimicrobial drugs within healthcare institutions is imperative to pinpoint actions essential for refining prescription appropriateness. These measures are critical in terms of well-being, presenting patients with optimal therapeutic options, consequently combating the development of resistance and diminishing associated unfavorable reactions. Additionally, they contribute to the economic viability.

## 2. Results

### 2.1. Data Collection

The results illustrated in the tables provide a detailed overview of the trend in the consumption of systemic antimicrobials in two hospital settings, Policlinico Umberto I in Rome and Azienda Sanitaria ASL Napoli 3 South. The extrapolated data exclusively pertain to hospital antibiotic consumption as this is a monitoring analysis that does not necessitate statistical tests with the inclusion of additional data. This study aims to raise awareness of the current usage of antimicrobials in Italy, utilizing two distinct Italian contexts as benchmarks. The intra- and inter-structure comparison assesses variability over time, among departments, and across different classes of antimicrobials. Table 1 reveals that in the first half of 2023, Policlinico Umberto I recorded a consumption of systemic antimicrobials (J01), expressed in Defined Daily Dose (DDD), amounting to 312,828.70. In particular, the indicators for consumption rates per 100 days of hospitalization (J01/G100 = A1) and per 100 discharges (J01/D100 = A2) were 211.3 and 2063.90, respectively. During the same period, Azienda Sanitaria ASL Napoli 3 South exhibited a different scenario, with a six-month DDD value of 63,671.86. The corresponding A1 and A2 indicators were 118.20 and 868.65, respectively. Among systemic antimicrobials, Penicillins and Beta-lactams (J01C), other Beta-lactams (J01D) not included in the previous group, and Cephalosporins (J01DD) constitute the most requested pharmacological classes in both structures. In this study, Policlinico Umberto I showed a higher consumption of the J01C class, as evidenced by the A4 indicator (Penicillins and Beta-lactams), approximately double that of ASL Napoli 3 South. Also, for the “other Beta-lactams” class (A5), albeit to a lesser extent, it was higher in the Roman reality (+29.27%); conversely, for Cephalosporins (Indicator A6), a positive trend reversal (+19.57%) was observed for Azienda ASL Napoli 3 South.

For the remaining classes of antimicrobials (from A7 to A15 and A3), consumption varies in the two healthcare companies when comparing the indicator values, both in cases at 100 days of discharges and 100 days of hospitalization. From the table, it is noteworthy that in the Roman hospital, the consumption of Carbapenems (A8) at 100 discharges was 6 times higher than that of the Campania company. High values of the A13 indicator describe for both structures sustained consumption at 100 discharges of the antimicrobial class of Fluoroquinolones, albeit with a trend reversal for ASL Napoli 3 South (+14.21%). The lowest and variable values were found for Tetracyclines (A3), Sulfonamides and Trimethoprim (A9), Cephalosporins IV gen. (A9BIS), and Colistin (A15) and were, respectively, 3.8, 3.4, 3.7, and 6.6 for Policlinico Umberto I and 0.64, 0.24, 0.34, and 0.74 for ASL Napoli 3 South. The A11 indicator for Quinolones also remained low for the Roman reality (2.7), while it differed for the Campania healthcare company (16.88). Finally, the consumption of the remaining classes of antimicrobials, Glycopeptides (A14), Carbapenems (A7), and Fluoroquinolones (A12, at 100 days of hospitalization), showed intermediate values of their respective indicators, depicting a scenario of Roman versus Campania reality, positive for the first two indicators (+83.89% and +77.68%) and negative for the third (−35.43%).

### 2.2. Comparison 2022 vs. 2023

In order to assess variations over time in the consumption of systemic antibiotics in the two healthcare companies, monitoring was conducted over a two-year period from 2022 to 2023 and the differentials were calculated for both healthcare companies (Table 2). 

The consumption of systemic antibiotics per 100 days of hospitalization (A1) showed an increase of 1.60% from 2022 to 2023 for Policlinico Umberto I, while a decrease of −7.40% (A2) was observed for the same facility. In contrast, positive values for both A1 and A2 were recorded for the Campania facility, specifically +29.00% and +28.00%, respectively. These results, except for indicator A2 at Policlinico Umberto I, describe an increasing trend during the examined period. A systematic analysis of various classes of antimicrobials in the Roman hospital showed significant increases in the consumption of 4th-generation Cephalosporins (A9BIS), Cephalosporins (A6), and other Beta-lactams (A5), respectively, of 30.40%, 15.50%, 10.10%, and decreases for the classes: Colistin (−30.70%), Tetracyclines (−21.60%), and Quinolones (−19.60%), from the first half of 2022 to the first half of 2023. In ASL Napoli 3 South, positive variations in the first half of 2023 compared to the previous period were recorded for Penicillins and Beta-lactams (+64.00%), other Beta-lactams (+42.00%), Carbapenems (+32.00% at 100 days of hospitalization, +31.00% at 100 discharges), and 4th-generation Cephalosporins (26.00%), while a decrease in consumption was associated with Sulfonamides and Trimethoprim (−103.00%), Tetracyclines (−54.00%), Macrolides, Lincosamides, and Streptogramins (−50.00%), and Colistin (−13.00%). Despite the increasing trend in consumption in all regions, the data showed a wide variability between antibiotic classes and structures. Fluoroquinolone antibiotics showed a slight increase despite safety concerns [10,11], probably due to their extreme effectiveness in urinary tract infections. On the other hand, a significant decrease was observed in the usage of antibiotics that have been in common clinical practice for a long time and now have modest therapeutic efficacy, influenced by bacterial resistance phenomena. It is evident that new-generation beta-lactam drugs demonstrate great therapeutic efficacy in a hospital setting, leading to a significant increase in their use in the two periods compared. This rise is a cause for serious concern within global scientific societies as increased consumption is often associated with growing microbial resistance, which risks giving rise to increasingly resistant bacteria without effective treatments [12]. 

### 2.3. Number of Discharges and Hospitalisations

To understand more critically and analytically the data on the consumption of various classes of systemic antibiotics and to delineate the areas of greater application, a study was conducted. This study involved the calculation and classification of the number of discharges and hospitalization days within both healthcare companies. Unlike the Campania reality, where data on discharges/hospitalizations were calculated and distributed among the various companies that were part of ASL Napoli 3 South, at Policlinico Umberto I, being a very extensive healthcare facility with 1250 beds, the total numbers of discharges/hospitalizations were calculated, classifying them by departments (Table 3). From a macroscopic point of view, there was an increase in terms of discharges and hospitalizations from 2022 to 2023, and this could positively impact the consumption not only of systemic antibiotics but, in general, all drugs used in a hospital setting. A systematic analysis highlighted that the highest number of hospitalizations/discharges was attributable to five macro areas, classified as follows: B: Cardiovascular (surgical and non-surgical), C: Anesthesia and Intensive Care, E: Infectious, pulmonary, and metabolic diseases (diabetology), F: Rheumatology, Nephrology, Geriatrics, and Internal Medicine, G: Urology, Gynecology and Obstetrics, Pediatrics, and Gastroenterology. In 2022, the highest number of hospitalizations was associated with categories C, E, and F, followed by G and B. The departments of Infectious/pulmonary/metabolic diseases (diabetes) represented those with the highest number of hospitalizations for the first halves of 2022–2023. In the examined timeline, an increase was recorded for all departments except: Rheumatology, Nephrology, Geriatrics, and Internal Medicine (−1892); Anesthesia and Intensive Care (−1447); and finally, Infectious/pulmonary/metabolic diseases (diabetes), which experienced a slight decrease (−369) despite having the highest number compared to other departments. The number of discharges also increased in the considered two-year period, going from 12,673 to 15,157. All departments contributed positively to these data except Hematology/Oncology/Dermatology (−32).

Finally, for ASL Napoli 3 South, a reclassification of data (discharges/hospitalization days) was carried out for hospital entities within the ASL area itself: hospitals in the Stabiese Area, Torre del Greco, Boscotrecase, Peninsula Sorrentina, and Nola Area (Table 4). In particular, hospitals with a higher weight in terms of consumption were presumably those with a higher number of discharges and hospitalization days. In 2022, the hospital/hospitals in the Nola Area (2407) had a greater impact in terms of the number of discharges compared to those in the Stabiese Area (2158) and Peninsula Sorrentina (1494), while the number of hospitalizations was higher in the Stabiese Area (16,455), followed by the Nola Area (12,781) and Peninsula Sorrentina. In 2023, the numbers of discharges and hospitalization days remained low for the hospitals in Torre del Greco and Boscotrecase, and, once again, the numbers of the other three entities considered had a greater impact. In particular, an increase in the number of discharges was observed for the Stabiese Area (+101) and Peninsula Sorrentina (+134), and a decrease was observed for the Nola Area (−271). Hospitalization days, compared to the previous year, underwent changes of +3.41% (Stabiese Area), +3.63% (Peninsula Sorrentina), and +5.66% (Nola Area). Despite the fact that these increases were recorded for both indicators (discharges/hospitalizations), the differences between the total in 2022 and 2023 were almost negligible. This is due to variations in numbers within the hospitals of Torre del Greco and Boscotrecase, which, although much lower than the other entities, have positive or negative changes that act as a lever, balancing the total value.

## 3. Discussion

This study provides a comprehensive overview of the utilization patterns of various systemic antibiotics in two distinct Italian healthcare contexts: one situated in Central Italy and the other in the Southern region. The systematic analysis underscores the considerable geographic diversity in systemic antibiotic usage. In fact, Policlinico Umberto I emerged as the facility with the highest systemic antibiotic consumption among the two healthcare entities, a trend which was consistent over the two-year period. These consumption patterns are justified by a higher volume of hospitalizations, primarily stemming from five macro areas: cardiovascular, intensive critical care, uro-gynecological, infectious diseases, and the combined rheumatology–geriatrics–nephrology–internal medicine area. In contrast, ASL Napoli 3 South, despite an overall lower consumption, exhibited increasing percentages from 2022 to 2023 across all antibiotic classes except Tetracyclines, Sulfonamides and Trimethoprim, and Macrolides, Lincosamides-, and Streptogramins. The significant consumption is chiefly attributed to the Stabiese Area, Peninsula Sorrentina, and Nola Area, exerting a more substantial impact on the number of hospitalizations within ASL Napoli 3 South. Beta-lactam antibiotics, including penicillins and cephalosporins, stand out as widely employed therapies in hospital settings, falling under the Watch group of the AWaRe classification [13,14,15]. The latest-generation molecules, recently introduced to the market, demonstrate broad-spectrum efficacy against various bacterial strains [16,17]. It is crucial to note that antibiotics from previous generations might still be recommended as first- or second-line therapeutic options, despite their higher potential for developing resistance. The World Health Organization (WHO) suggests maintaining the priority of such drugs in local and national management programs. Projections on a broader scale indicate an upsurge in deaths due to infectious diseases in the absence of viable therapeutic alternatives, creating a critical scenario with limited means to counteract. In response, the Campania Region has implemented multiple actions, including the creation of new territorial organizations for physicians through the Territorial Functional Aggregation (TFA), aiming to establish a network among physicians for therapeutic continuity and continuous training [18,19,20]. This study delves into antibiotic usage in hospital departments, evaluating consumption rates through standardized parameters and indicators based on dispensed DDDs. The results highlighted an increase in antibiotic use in Italian hospitals, validated by the outcomes of Policlinico Umberto I in Rome. This increase, occurring in the post-COVID-19 pandemic period, contrasts with global campaigns to reduce consumption and preserve antimicrobial efficacy. Assessing and monitoring antibiotic prescriptions is crucial to manage antibiotic resistance in hospitalized patients [21,22,23,24,25,26,27]. Studies in the literature demonstrate that limiting antibiotic use to clinical cases requiring specific anti-infective interventions is essential to counteract antibiotic resistance, leading to fewer therapeutic failures. Periodic reports from public clinical and microbiological laboratories can be instrumental in promptly highlighting and informing affected individuals about detected bacterial infections. For instance, prescribing antibiotics based on oropharyngeal swabs, antibiograms, etc., ensures greater therapeutic appropriateness, ensuring the safety and efficacy of treatments while reducing antibiotic resistance. Rapid identification of the bacterial infection source allows targeted interventions on the transmission focus with high precision and accuracy, based on the spectrum of action and characteristics of the most suitable antibiotic [28,29,30]. Regarding healthcare-associated infections (HAIs), many infections are contracted primarily in healthcare settings, highlighting the inadequacy of current measures in eliminating or at least reducing them. HAIs affect immunocompromised patients, making them more vulnerable to bacterial infections and superinfections. Raising awareness among healthcare workers is crucial to ensure their utmost attention and increase treatment appropriateness. The results of this study can be instrumental in strengthening Antimicrobial Stewardship Programs (ASPs) and motivating other hospitals nationwide to measure their antibiotic consumption rates. The measurement and reporting of antibiotic consumption are integral components of ASPs. Implementing ASPs in hospitals has been shown to reduce antibiotic consumption rates. Furthermore, promoting awareness among healthcare staff through educational events, mandatory conferences, and internal training courses within hospital facilities is crucial for effective action against drug resistance and improved antibiotic utilization. The theme of health and socio-health prevention warrants special attention. Healthcare professionals might not be familiar with health disinfection, necessitating the emphasis on suitable products for the disinfection and sterilization of individuals, environments, and healthcare equipment. Specialized prevention courses are essential to support healthcare workers and patients in working safely. Additionally, several strategic actions have been activated within departments to enhance the prescription-dispensation and administration processes of antibiotics [31,32,33,34].

### Strengths and Limitations

The exhaustive data compilation process, encompassing diverse data sources such as national health databases, stand out as a noteworthy aspect. Additionally, the utilization of Defined Daily Doses (DDDs) as a standardized metric provides a means to compare antibiotic consumption across regions and countries. This method approximates the proportion of the population undergoing daily treatment with specific antibiotics, facilitating meaningful cross-regional and international comparisons. On the other side, the retrospective study design introduces potential limitations related to data availability, accuracy, and the presence of confounding factors not considered during data collection. The reliability of the findings may be influenced by variations in data collection methods and potential biases in reporting observed across diverse secondary data sources.

## 4. Methods

Reports on the first half of 2023 regarding the utilization of antimicrobial drugs in a hospital setting (ATC J01) have been assessed in two different Italian contexts. These contexts are located in two distinct Italian regions and differ because one is a university hospital (AO), whereas the second is a local health authority (ASL) made up of five small community hospitals. The Policlinico Umberto I in Rome is a university hospital with 1250 beds, accommodating 20,000 daily visitors, and stands among the largest hospitals in Rome, the Italian capital in the Lazio region. Azienda Sanitaria ASL Napoli 3 South is one of the health companies in the Campania region, consisting of five hospitals (with approximately 850 beds) and ten health districts, serving a population of over one million. In the hospital context, when comparing drug consumption between different departments or hospitals or during different periods, the use of indicators such as the number of Defined Daily Doses (DDDs) per 100 regular hospitalization days and the number of DDDs per 100 regular discharges is recommended. For the analysis, data from the “Hospital Consumption File” have been used to account for drug consumption in company structures for the New health/sanitation information system (NSIS), along with data related to hospitalization days and the number of discharges from the company’s “Hospital Discharge Forms” registration system [35,36]. In both health companies, all data have been extracted using internal management software with control of information flows recorded at the time of hospitalization/discharge. The extraction process considered the following parameters: quantity of consumed medicine expressed in the smallest unit of measure, such as tablets or vials, and the utilizing department for AO. For the reference community hospital for ASL, they were the number of hospitalization days for each department/hospital; the number of discharges for each department/hospital. After extraction, an Excel file has been created, incorporating all values obtained from the extraction. A column has been dedicated to the Defined Daily Dose (DDD) for the molecules under evaluation and the ATC classification, based on the WHOCC (WHO Collaborating Centre for Drug Statistics Methodology) values updated to October 2023. Subsequently, indicators of interest were calculated according to the required parameters (Table 5), creating a table displaying the DDDs for the first half of 2023 and the corresponding indicator value, calculated based on the previously created indicator table. Another table has been generated to compare the indicators between the first half (1H: January–June) of 2022 and 2023, including a column for percentage variation. One final table was created to compare the number of discharges and hospitalization days between 1st January and 30th June of the year 2022 and 2023, broken down by departments/hospitals.

## 5. Conclusions

In the aftermath of the pandemic, the main threat to global public health is antibiotic resistance. Consequently, several national initiatives, led by the WHO, have been launched to tackle antimicrobial resistance, with the aim of promoting informed and appropriate use of antibiotics. The main driver of microbial resistance is the excessive and inappropriate consumption of antibiotics. Without a real change towards a prudent use of antimicrobials, this resistance may lead to inevitable deaths as effective drugs are in short supply among current treatment options. The analysis of real-world evidence clearly indicates a significant post-pandemic surge in antibiotic use in the Italian context, which serves as the baseline for this study. In particular, the most recent antibiotics introduced on the market, such as penicillins and latest-generation cephalosporins, are used overwhelmingly. This trend probably highlights the limited effectiveness of traditional antibiotics in the hospital setting. It is essential to establish routine monitoring of consumption and expenditure in healthcare facilities [37,38,39]. This practice ensures that healthcare personnel are well informed about the current emergency situation, while, at the same time, reinforcing awareness-raising campaigns to steer towards a more targeted and appropriate use of available drugs.

## Figures and Tables

**Table 1:** DDD values calculated for the various classes of systemic antibiotics at the Policlinico Umberto I hospital and ASL Napoli 3 South.

		AO Policlinico Umberto 1		ASL Napoli 3 South	
Indicator	Description Indicator	DDD per 100 Regular Hospitalization Days 1H 2023	Indicator	DDD per 100 Regular Hospitalization Days 1H 2023	Indicator
A1	rates of J01 per 100 hospital days in ordinary regimen	312,828.70	211.30	63,671.86	118.20
A2	J01 rates per 100 ordinary discharges	312,828.70	2063.90	63,671.86	868.65
A3	tetracycline rates (J01A) per 100 hospital days	5628.00	3.80	346.50	0.64
A4	rates of penicillins and betalactams (J01C) per 100 hospital days	84,013.70	56.70	15,781.48	29.30
A5	rates of other betalactams (J01D) per 100 hospital days	91,294.90	61.70	23,509.25	43.64
A6	rates of cephalosporins (J01DD) per 100 hospital days	32,099.40	21.70	14,531.58	26.98
A7	rates of carbapenems (J01DH) per 100 hospital days	28,768.90	19.40	2331.00	4.33
A8	carbapenemi (J01DH) rates per 100 discharges	28,768.90	189.80	2331.00	31.80
A9	rates of sulphonamides and trimethoprim (J01EE) per 100 hospital days	5010.00	3.40	126.99	0.24
A9BIS	rates of cephalosporins IV gen. (J01DE) per 100 hospital days	5466.50	3.70	185.00	0.34
A10	rates of macrolides, lincosamides and streprogramins (J01F) per 100 hospital days	23,407.30	15.80	7790.17	14.46
A11	rates of quinolones (J01M) per 100 hospital days	3990.50	2.70	9091.50	16.88
A12	fluoroquinolone (J01MA) rates per 100 hospital days	16,133.00	10.90	9091.50	16.88
A13	rates of fluoroquinolones (J01MA) per 100 discharges	16,133.00	106.40	9091.50	124.03
A14	glycopeptide rates (J01XA) per 100 hospital days	18,704.50	12.60	1092.50	2.03
A15	colistin rates (J01XB01) per 100 hospital days	9792.50	6.60	398.64	0.74

**Table 2:** DDD values, over the two-year period 2022–2023, for the various classes of systemic antibiotics at the Policlinico Umberto I hospital and ASL Napoli 3 South.

		AO Policlinico Umberto 1			ASL Napoli 3 South		
Indicator	Description Indicator	Indicator	Indicator	∆%	Indicator	Indicator	∆%
		1H 2022	1H 2023		1H 2022	1H 2023	
A1	rates OF J01 per 100 hospital days in ordinary regimen	207.90	211.3	1.60%	83.62	118.20	29%
A2	J01 rates per 100 ordinary discharges	2228.10	2063.90	−7.40%	624.68	868.65	28%
A3	tetracycline rates (J01A) per 100 hospital days	4.80	3.80	−21.60%	0.99	0.64	−54%
A4	rates of penicillins and betalactams (J01C) per 100 hospital days	52.80	56.70	7.50%	10.60	29.30	64%
A5	rates of other betalactams (J01D) per 100 hospital days	56	61.70	10.10%	25.44	43.64	42%
A6	rates of cephalosporins (J01DD) per 100 hospital days	18.80	21.70	15.50%	22.17	26.98	18%
A7	rates of carbapenems (J01DH) per 100 hospital days	19	19.40	2.30%	2.96	4.33	32%
A8	carbapenemi (J01DH) rates per 100 discharges	203.50	189.80	−6.70%	22.08	31.80	31%
A9	rates of sulphonamides and trimethoprim (J01EE) per 100 hospital days	3.20	3.40	5.90%	0.48	0.24	−103%
A9BIS	rates of cephalosporins IV gen. (J01DE) per 100 hospital days	2.80	3.70	30.40%	0.26	0.34	26%
A10	rates of macrolides, lincosamides and streprogramins (J01F) per 100 hospital days	15.90	15.80	−0.60%	21.68	14.46	−50%
A11	rates of quinolones (J01M) per 100 hospital days	3.40	2.70	−19.60%	15.30	16.88	9%
A12	fluoroquinolone (J01MA) rates per 100 hospital days	11	10.90	−0.70%	15.30	16.88	9%
A13	rates of fluoroquinolones (J01MA) per 100 discharges	117.60	106.40	−9.50%	114.30	124.03	8%
A14	glycopeptide rates (J01XA) per 100 hospital days	14.50	12.60	−12.70%	1.76	2.03	13%
A15	colistin rates (J01XB01) per 100 hospital days	9.50	6.60	−30.70%	0.83	0.74	−13%

**Table 3:** Number of discharges and hospitalizations calculated for Policlinico Umberto I over the two-year period 2022–2023.

Departments		1H 2022 (Indicator)		1H 2023 (Indicator)	
		n. Resigned	GG Hospitalisation	n. Resigned	GG Hospitalisation
A	Laparoscopic, orthopedic and trauma, plastic and breast surgery	1881	11,965	2327	14,285
B	Cardiology, vascular surgery, NICU, cardiac surgery	1707	17,982	2041	18,078
C	Anaesthesia and resuscitation, intensive care, and emergency surgery	1262	23,513	1271	22,066
D	Haematology, oncology, and dermatology	366	5892	334	6072
E	Infectious diseases, diabetes, pulmonology, and cystic fibrosis	1358	24,694	1421	24,325
F	Dialysis and nephrology, geriatrics, rheumatology, and internal medicine	1240	22,646	1254	20,754
G	Urology, gynaecology and obstetrics, paediatrics, and gastroenterology	3630	18,364	3960	20,299
H	Neurosurgery, neurology, and psychiatry	1229	15,157	1532	17,337
I	Otolaryngology, ophthalmology, dentistry, maxillo-facial surgery	762	3753	1017	4852
	**Total**	**12,673**	**143,966**	**15,157**	**148,068**

**Table 4:** Number of discharges and hospitalizations calculated over the two-year period 2022–2023 for ASL Napoli 3 South.

Hospital	1H 2022 (Indicator)		1H 2023 (Indicator)	
	n. Resigned	GG Hospitalisation	n. Resigned	GG Hospitalisation
Area Stabiese	2158	16,455	2259	17,036
Torre del Greco	640	6014	470	4565
Boscotrecase	663	9130	837	7638
Penisola Sorrentina	1494	10,678	1628	11,080
Area Nolana	2407	12,781	2136	13,548
**Total**	**7213**	**53,884**	**7330**	**53,867**

**Table 5:** Indicators used for analyzing and monitoring antibiotic consumption in hospital settings.

Cod. Indicator	Description Indicator
A1	rates of J01 per 100 hospital days in ordinary regimen
A2	J01 rates per 100 ordinary discharges
A3	tetracycline rates (J01A) per 100 hospital days
A4	rates of penicillins and betalactams (J01C) per 100 hospital days
A5	rates of other betalactams (J01D) per 100 hospital days
A6	rates of cephalosporins (J01DD) per 100 hospital days
A7	rates of carbapenems (J01DH) per 100 hospital days
A8	carbapenemi (J01DH) rates per 100 discharges
A9	rates of sulphonamides and trimethoprim (J01EE) per 100 hospital days
A9BIS	rates of cephalosporins IV gen. (J01DE) per 100 hospital days
A10	rates of macrolides, lincosamides and streprogramins (J01F) per 100 hospital days
A11	rates of quinolones (J01M) per 100 hospital days
A12	fluoroquinolone (J01MA) rates per 100 hospital days
A13	rates of fluoroquinolones (J01MA) per 100 discharges
A14	glycopeptide rates (J01XA) per 100 hospital days
A15	colistin rates (J01XB01) per 100 hospital days

## Data Availability

Full availability of data and materials. All stated data can be provided on request to the reader.
